# Blood Pressure in Old Population

**DOI:** 10.1155/2012/610525

**Published:** 2012-02-23

**Authors:** Blas Gil-Extremera, Jaako Tuomilehto, Pedro Cía-Gómez

**Affiliations:** ^1^Service of Internal Medicine, San Cecilio Universitary Hospital, University of Granada, 18012 Granada, Spain; ^2^Department of Public Health, University of Helsinki, 00014 Helsinki, Finland; ^3^Welfare and Health Promotion Division, National Institute for Health and Welfare, 00300 Helsinki, Finland; ^4^Diabetes Prevention Unit, Department of Chronic Disease Prevention, P.O. Box 30, 00271 Helsinki, Finland; ^5^Department of Internal Medicine, “Lozano Blesa” Clinical University Hospital, University of Zaragoza, 15 Avenida San Juan Bosco, 50009 Zaragoza, Spain

This monograph issue of the journal is dedicated to the most frequent cardiovascular disorders in the Western countries: hypertension, mostly in the elderly. In this situation, two patterns can be found: (a) increased systolic and diastolic blood pressure and (b) isolated systolic hypertension.

A total of fourteen selected papers are included; nine of them are about clinical aspects of the disease and the remaining about pathophysiological profile of hypertension in old population.

The papers by A. Paganini-Hill and M. Igase et al. of this issue address the relationship between hypertension and dementia. The authors showed the deleterious role of cardiovascular risk factors—mostly increased blood pressure—on the incidence of dementia; 13978 old people (mean age 74 years old) after followup from 1981 to 2010 were studied. In summary, high blood pressure and its treatment have different effects in men and women in the elderly. The second one also establishes the relationship between hypertension and dementia; recent clinical trials suggest that blockades of RAS system could have reduced cognitive decline seen in Alzheimer's disease and vascular dementia. The paper of B. Gil-Extremera1 and P. Cía-Gómez is a background, by the clinical point of view, of hypertension in older population with special attention to the combination therapy of hypertension with more benefits than monotherapy. The study done by A. Siennicki-Lantz and S. Elmståhl concluding: “hypertension and vascular risk factors in a cohort of 68-years-old men do not result in higher ambulatory blood pressure monitoring (ABPM) at age 82, possibly due to inflection point in their pressure development” [sic]. The next one study analyses ABPM in the elderly, because the prevalence of hypertension by AMBP is not well known in this particular population. The paper by N. K. Patel et al. highlights the relevance of effectiveness, culturally responsive hypertension management among the high-risk Hispanic patients for achieving positive health results. The paper by B. Gil-Extremera reports some ideas concerning the clinical experience of the author on lipid disorders and some frequent errors observed in his clinical practice; for example, some physicians take as normal lipid values clearly high, and many patients do not receive any treatment; statin/fibrate combination therapy is not recommended, but unfortunately this procedure is maintained in many cases; another common error is the discontinuation of lipid lowering therapy when normal values are reached.

Other two papers raise the benefit of antihypertensive treatment on very old hypertensive patients according to the HYVET Study, and one of them reports that control of elderly hypertension in sub-Saharan Africans was effective and required at least two antihypertensive drugs.

Additional five papers belong to the experimental or basic aspects of hypertension; the role of C-reactive protein in atherosclerosis, the mechanisms related to hypertension in the elderly, the biochemical and molecular aspects of vascular adrenergic regulation of blood pressure, the putative role of protein Klotho in cardiovascular and renal disease, and, finally, the role of cortisol secretions in hypertensive elderly patients.

*Blas Gil-Extremera* *Blas Gil-Extremera* *JaakoTuomilehto* *JaakoTuomilehto* *Pedro Cía-Gómez* *Pedro Cía-Gómez*

